# IVF and ICSI in Male Infertility: Update on Outcomes, Risks, and Costs

**DOI:** 10.1100/tsw.2005.117

**Published:** 2005-11-16

**Authors:** Edward Karpman, Daniel H. Williams, Larry I. Lipshultz

**Affiliations:** Scott Department of Urology, Baylor College of Medicine, Houston, TX 77030, USA

**Keywords:** Intracytoplasmic sperm injections, in vitro fertilization, male infertility

## Abstract

Assisted reproductive technology with intracytoplasmic sperm injection (ICSI) is becoming an international panacea for couples struggling with infertility. The increasing popularity of these techniques and the data generated has given us a better understanding of the efficacy, consequences and costs of these procedures. There still remain many unanswered questions and controversies surrounding the use of IVF and ICSI. Increased experience, better refinement of these techniques and clearer indications for IVF and ICSI will inevitably minimize the risks associated with this procedure.

## INTRODUCTION

Infertility is becoming an increasing problem in the U.S. Approximately one out of six couples will have difficulty initiating a pregnancy. Problems with conception are related to a significant female factor in approximately 50% of the cases, a significant male factor in 30%, and a combined male/female factor in another 20% of cases[1].

Historically, infertility specialists have had great success in treating obstructive azoospermia, varicoceles, ovulatory dysfunction, and cervical factor abnormalities as causes of infertility. In the past, however, nonobstructive etiologies such as azoospermia, severe oligoasthenoteratozoospermia, or nonreconstructable ductal obstruction essentially precluded a couple from having their own genetic children. The limited options for such couples were either donor sperm insemination or adoption.

The birth of Louise Brown in 1978 using *in vitro* fertilization (IVF) ushered in a new era of reproductive medicine that has given thousands of couples the opportunity to conceive their own genetic offspring. IVF was initially used for the treatment of fallopian tube obstruction. However, it quickly became apparent that this innovative technique could be used for many other causes of both male and female infertility. Almost by accident, the discovery by Palermo and colleagues in 1992 that a single sperm injected into a human egg could result in a viable embryo further revolutionized the field of assisted reproductive technology (ART)[2]. Intracytoplasmic sperm injection (ICSI) allowed even men with otherwise untreatable reproductive failure to conceive their own offspring. By 1993, ICSI was already being offered to patients at IVF centers across the U.S. The remarkable success of ICSI decreased the number of subfertile couples who remained involuntarily childless to only 4%[3].

Despite the immediate success and widespread use of ICSI, there was no vigorous internal review board (IRB) approval; an ethics committee did not deliberate the biological impact of ICSI, nor did extensive animal research antedate its use in humans. IVF/ICSI was offered to thousands of patients without any government oversight or regulatory action by the Food and Drug Administration (FDA). It was even offered to couples with reproductive tract obstructions, bypassing the opportunity for reconstruction and spontaneous pregnancy and disregarding the additional costs of this technique[4]. Only recently have several large multicenter international studies questioned the risks that might be associated with ICSI.

In the past, the success of IVF and ICSI was only evident from published reports from individual large centers[5,6,7]. The outcomes from the majority of IVF centers in the U.S. were never made public for scrutiny and comparison. It was not until the establishment of the Society for Assisted Reproductive Technology (SART) and the passage of a law by Congress, the Fertility Clinic Success Rate and Certification Act, that consumers were able to evaluate the success rates of individual centers[8]. In 1995, the Centers for Disease Control and Prevention (CDC) collaborated with SART and took on the role of publishing the clinical outcomes from the vast majority of fertility clinics providing ART in the U.S. These data are now readily available and allow us to assess the true success and impact of IVF and ICSI.

## OUTCOMES

In 2002, there were 115,392 ART procedures reported to the CDC; IVF and IVF/ICSI comprised 46.3 and 52.8% of all fresh, nondonor egg procedures, respectively[9]. Less than 1% of the remaining cycles employed other ART techniques. This number of total ART cycles represents an increase of 7.2% from the previous year. When evaluating ART data, there are several reportable end points for success and include rates of fertilization, number of embryos transferred, clinical pregnancies, and live birth rates achieved. It is important to remember that, for the consumer, the “take-home baby rate” is all that matters. The live birth rate per cycle for IVF and IVF/ICSI was 34 and 31.9%, respectively. Stratification of the data into quartiles for high-volume and low-volume centers demonstrated no difference in the rate of live births per cycle between those that performed less than 61 or greater than 251 cycles per year (27.4 vs. 28%, respectively).

Patient characteristics, sperm characteristics, etiology of infertility, method of sperm retrieval, and technique of sperm processing have all been evaluated as predictors of success with IVF and IVF/ICSI. The impact of increasing maternal age is a well-known negative predictor of successful spontaneous pregnancy[10]. The data from the CDC demonstrate that advancing maternal age over 35 years has a significant and progressive impact on live birth rates per cycle of IVF and IVF/ICSI (Fig. 1). Multiple reports from different countries have demonstrated similar outcomes[11]. These data would support maternal age as the single-most important predictor for having a successful outcome in IVF and ICSI.

Other patient characteristics such as paternal age and female obesity have also been evaluated. It is well known that there are significant hormonal alterations with both aging and obesity. Longitudinal studies show a decrease in testosterone starting in the early 40s in men and gradually worsening through the remainder of life[12]. Semen volume, total sperm production, and normal sperm morphology were all found to be lower in men 52–79 as compared to a control group younger than 52[13]. Despite these findings, increasing paternal age was previously shown not to impact on IVF or IVF/ICSI success rates[7]. However, a more recent prospective study has brought into question the role of advancing paternal age on IVF success[14]. In this study by Klonoff-Cohen and Natarajan, sperm number, pregnancy, and live birth delivery were all inversely related to increasing paternal age. Obesity alters relative ratios of testosterone (T) and estradiol (E2) in both sexes due to peripheral conversion of T to E2 by aromatase. Despite this, maternal obesity (BMI > 27) does not seem to impact clinical pregnancy rates based on the results of one large retrospective study evaluating “good-prognosis patients”[15].

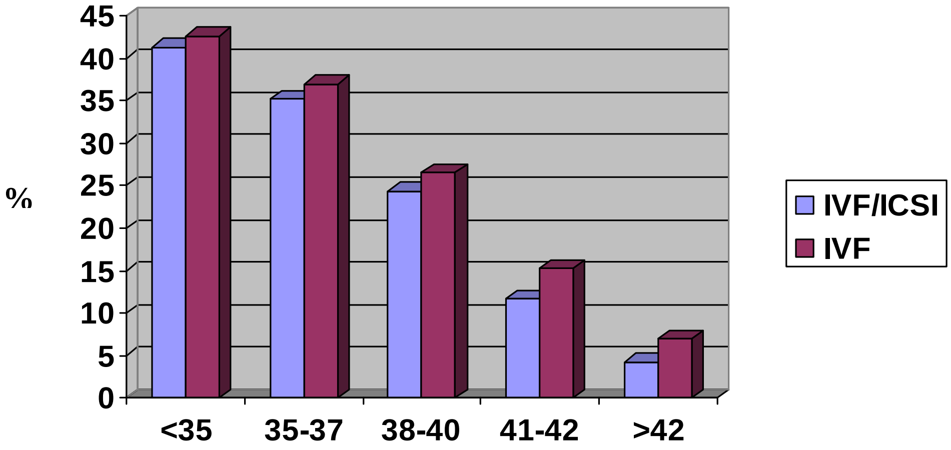

**FIGURE 1**. Percent live births per retrieval by woman's age (from 2002 CDC/SART data)

The application of IVF/ICSI is now heavily focused on those couples with severe male factor infertility (i.e., nonobstructive azoospermia [NOA] and severe oligoasthenoteratozoospermia [OATS]). The assisted use of a single spermatozoa to fertilize an oocyte obviates the need to improve sperm density significantly in many cases. It is no surprise, therefore, that researchers have not found a correlation between sperm density and success rates in IVF/ICSI[16]. In contrast, sperm motility and strict morphology determinants have been reported, albeit with some controversy, to impair IVF/ICSI success[6,17,18,19,20,21].

The correlation between morphology (Tygerberg strict criteria) and conventional IVF success rates has clearly been established[22,23,24,25]. Semen samples with less than 14% normal morphology had a significantly lower fertilization rate (37–47%) than the groups with greater than 14% normal morphology (85–88%). It has been postulated that normal sperm morphology is important for sperm adherence to aid penetration of the zona pellucida. Injecting a single spermatozoa into the ooplasm with ICSI bypasses this step in normal fertilization. Numerous studies have shown that fertilization, embryo transfer, and pregnancy are not related to sperm morphology following ICSI[6,16,17,18,19]. Additionally, other investigators have shown that pregnancy outcome and newborn/infant status of couples using ICSI with profound teratospermia (0% normal morphology) was similar to controls having strict morphology greater than zero[20]. In this study by McKenzie et al., there were 21 pregnancies in 45 couples with an average gestational age of 38.4 weeks. There were no birth defects noted at delivery and all children reached their developmental milestones at a mean 15 months of follow-up. Despite all of these encouraging data minimizing the role of sperm morphology in IVF/ICSI success, there is a more practical side to this matter. Although only one sperm is required to fertilize an oocyte, numerous sperm are usually available for the embryologist working with the sperm sample and only the “best looking” sperm are selected for fertilization. Therefore, a more appropriate assessment of the relationship between sperm morphology and IVF/ICSI success would be based on the evaluation of the single sperm injected. When looking at the individual sperm morphology, De Vos et al. found that injection of morphologically abnormal sperm resulted in lower fertilization rates (71.7 vs. 60.7%), pregnancy (36.7 vs. 18.7%), and implantation rates (20.2 vs. 9.6%)[21]. The lower success rates using morphologically abnormal sperm for ICSI is further supported by evaluating the rate of aneuploidy in these cells. Ryu et al. studied the rate of aneuploidy of the sex chromosomes and chromosome 18 using fluorescence *in situ* hybridization (FISH) in men undergoing ICSI[26]. They found that morphologically abnormal sperm had a mean aneuploidy rate of 29% compared to 1.8–5.5% in the morphologically normal sperm from the same specimen.

A prerequisite for the success of ICSI is the use of a viable sperm that will support embryogenesis after microinjection. Motile sperm are an absolute sign of viability, but immotile sperm do not necessarily represent a dead cell, particularly when retrieved from the testis. Fertilization and pregnancy rates were significantly improved in both fresh and frozen-thawed sperm from testicular sperm extraction (TESE) samples when motile were compared to immotile sperm during ICSI[27,28]. In the absence of motility, some authors have demonstrated alternative ways of demonstrating viability. Traditionally, the hypo-osmotic swelling (HOS) testing has been found to be a useful predictor of sperm viability in immotile sperm and correlates well with fertilization and pregnancy rates without damaging sperm[27,28,29]. Incubating sperm in pentoxifylline-enriched media and a mechanical “touch” technique demonstrating coiling and uncoiling of the tail of sperm have also been reported tests for determining sperm viability[30,31].

Prior to the advent of ICSI, patients with NOA or OA were left with minimal options for conceiving their own genetic offspring. Even if sperm were found in the epididymis or testis, the success of conventional IVF in fertilizing oocytes was extremely poor. Silber et al. reported a fertilization and take-home baby rate of 19 vs. 90% and 4.5 vs. 42%, respectively, in men with OA undergoing microsurgical epididymal sperm aspiration (MESA) and conventional IVF or MESA/ICSI[32]. Results such as these emphasized the significance of sperm maturation and processing through the male reproductive tract as important steps for natural sperm/oocyte penetration. However, since even patients with severe OATS have adequate numbers of spermatozoa in their ejaculate to undergo ICSI, the question then became whether the source of sperm (ejaculated, epididymal, or testicular) made a difference for predicting successful outcomes with ICSI. In one retrospective study addressing this issue, the investigators found that ejaculated sperm from patients with male factor infertility, epididymal sperm form OA patients, and testicular sperm from OA patients had similar rates of fertilization and pregnancy[33]. Futhermore, these authors found that patients with testicular sperm from NOA patients had the worst fertilization and pregnancy rates, while patients with ejaculated normal semen analyses, but a tubal factor, had the best overall success rates. The debate of whether the outcomes with ICSI differed when using epididymal or testicular sperm persisted in the literature for several years. The results from a recent meta-analysis evaluating a total of 734 cycles of ICSI with either epididymal or testicular sperm found no difference in cleavage, pregnancy rate, or implantation rate among the two groups[34]. However, when these authors compared the results of 1,103 ICSI cycles in patients with OA and NOA, there was a statistically significant improvement in fertilization (RR 1.18) and clinical pregnancy rates (RR 1.36) in the OA group. Collectively, these data would suggest that the source of sperm harvesting is not as important as is the underlying etiology of infertility.

Pretreatment cryopreservation of sperm has been utilized extensively in fertility clinics for overcoming the damaging effects of surgery, chemotherapy, and radiation treatment[35]. In the era of ICSI, cryopreservation has been utilized to have extracted sperm available prior to female ovarian induction and oocyte retrieval and to perform, if necessary, repeated cycles of IVF/ICSI without the need for multiple surgical procedures for sperm procurement. Despite the availability of cryopreservation of sperm and the convenience that it offers to the patient, sperm banking can significantly reduce the number of motile sperm after thawing[36]. This concern is most pronounced in patients undergoing TESE for NOA because of the inherently lower number and poorer quality of sperm that are retrieved during this procedure[30,37]. Conflicting reports about the success of using fresh or frozen sperm from both the epididymis and testis for ICSI have been reported in the literature. The results from a recent meta-analysis demonstrated an increased pregnancy rate when using fresh sperm[34]. However, when the data were further analyzed for epididymal and testicular samples, these results were only supported for epididymal and not testicular sperm.

## RISKS

Concerns regarding ICSI have been present since the inception of this procedure. The procedure was quickly brought to market without extensive evaluation of its biological impact. Critics argued that by bypassing the natural selection of sperm-zona penetration, we would increase the risk of severe malformations in the offspring. Initial small prospective and retrospective reports did not seem to support this concern[38,39,40]. However, it has now been almost 13 years since the first ICSI babies were born and larger studies comparing 5-year-old ICSI babies to natural conception (NC) controls have shed more light on the subject.

It is important to remember that a multiple gestation pregnancy (MGP) is a common occurrence with ART. During any cycle of IVF or IVF/ICSI, multiple embryos are transferred simultaneously in order to maximize a high success rate. This practice results in a 20-fold increased risk of twins and a 400-fold increased risk of higher-order pregnancies as compared to NC[41]. Multiple gestations can be a welcome occurrence for many couples, but this end result is associated with an increased risk of premature labor, low or very low birth weight, and perinatal death[42,43,44,45]. According to the 2002 SART/CDC data, approximately 29% of all IVF and ICSI cycles resulted in a twin gestation and an additional 7% of cycles were reported to be triplets or higher order[9]. The live birth rate of twins and higher order gestation was 32 and 4%, respectively. This discrepancy is due to stillborn births (6%), natural fetal death, and selective multiple pregnancy reduction, which was much more common in triplet and higher-order gestations[9]. Because of the prevalence of MGP and the associated risks to the fetus and mother, recent efforts by fertility centers to reduce the number of embryos transferred have been initiated. SART has recently established guidelines for the transfer of embryos[46,47]. In women less than 35 years of age with good quality embryos and undergoing a first cycle of IVF, no more than two embryos should be transferred. The opposite end of the spectrum includes women older than 40 years old who can have up to five embryos transferred per cycle. These are only guidelines and no requirements have been imposed on individual fertility centers. Other countries' attempts to reduce MGP have been reported. Using the database established by the Human Fertilization and Embryology Authority in the U.K., Templeton et al. showed that the success rate was no greater for three vs. two transferred embryos and this reduction essentially eliminated the risk of higher order MGP[48]. However, the twin pregnancy rate remained the same. In another prospective multicenter trial, good-prognosis patients (women less than 36 years old and at least two good-quality embryos) were randomized to either double-embryo transfer (DET) or single-embryo transfer (SET) with additional frozen-thawed SET if necessary[49]. This technique reduced the MGP from 33.1 to 0.8%. However, this technique also reduced the live birth rate from 42.9 to 27.6% for the SET cycle. The results of using SET are slightly worse when using unselected patients. A recent randomized study from the Netherlands comparing SET to DET in *unselected* patients found an ongoing pregnancy rate of 21.4 vs. 40.3% and a twin pregnancy rate of 0 vs. 21%, respectively[50]. In countries like the U.S., where the financial burden of ART is the responsibility of the patient, decreasing the success of IVF/ICSI by almost 50% may be cost prohibitive.

Early reports dealing with the risk of congenital malformations and psychological development of children born after ICSI did not reveal any differences as compared to naturally conceived children[2,38,40]. Subsequent studies identified a higher risk of hypospadias in IVF offspring[51,52]. This increased congenital defect was attributed to paternal subfertility and implied a genetic etiology. Using the statutory Reproductive Technology Register in Western Australia, in one of the first large retrospective studies, Hansen et al. demonstrated that children born after IVF and IVF/ICSI and examined at 1 year of age had more major and minor birth defects than NC controls[53]. These authors found a greater than twofold risk of birth defects in both conventional IVF (9.0%) and IVF/ICSI (8.6%) children compared to controls (4.2%). There was a higher risk of chromosomal defects, musculoskeletal defects, and multiple major defects in the ICSI cohort compared to the NC controls. These differences remained significant even when controlling for singleton infants, infants born at term (greater than 37 weeks), maternal age, and parity. This study was criticized for its retrospective nature and for using a birth defect registry to identify these children. However, a subsequent report from the German ICSI Follow-Up Study

Group prospectively evaluated ICSI children (N = 3,372) and compared them to matched controls (N = 8,016)[54]. All children were evaluated within 6 weeks of birth by experienced neonatologists and medical genetics experts as well as utilizing sonography of the hips, kidney, and urinary tract. A significant increase in major malformations of the heart, GI tract, and urinary tract, as well as chromosomal abnormalities, was seen in the ICSI infants as compared to controls. Additionally, when looking at perinatal outcomes of singleton pregnancies, there was a significant difference in gestational age, birth weight, body length, head circumference, and APGAR scores in the ICSI children compared to NC controls. Most recently, these differences have been confirmed by the International Collaborative Study of ICSI-Child and Family Outcomes (ICSI-CFO) study[55]. Detailed assessment of 5-year-old ICSI singleton children born at greater than 32 weeks gestation were matched by age, sex, maternal education, and socioeconomic status to controls. The multinational analysis showed a significant increase in pregnancy complications, neonatal unit admissions, and admissions to the hospital greater than 7 days for ICSI children compared to controls. Additionally, there was a greater incidence of significant childhood illnesses in ICSI children (74 vs. 57%). ICSI children had a greater likelihood of requiring physiotherapy, speech/language therapy, acquiring upper and lower respiratory tract infections, having dermatological or gastrointestinal problems, or requiring any type of surgery than NC controls. Collectively, the results of several of these studies have been analyzed and the results suggest a statistically significant increased risk of 30–40% of birth defects associated with ART[56].

Previous reports have implicated the procedure of IVF/ICSI itself in the increased risk of low-birth-weight babies, premature births, and neurologic sequelae. A recent report has shown that approximately 10.4% of IVF singletons originated from a twin gestation early in pregnancy[57]. The authors found that these infants from a vanishing twin pregnancy carried a greater risk of birth defects than their singleton counterparts. These findings would support the use of SET in reducing some of the risks associated with IVF/ICSI, despite the additional costs.

The association between genetic abnormalities and male infertility has been well known for many years, but this association is primarily seen in men with azoospermia or severe oligozoospermia (less than 5 million/ml). Azoospermia is reported in approximately 10–20% of patients referred to an infertility clinic with approcimately 63% of those being due to testicular failure[58]. Infertility represents a natural selection process where defective genes are not propagated in the population gene pool by preventing fertilization of the oocyte. The advent of ICSI bypasses this natural selection process and has allowed couples with even the most severe cases of genetic infertility to procreate.

Autosomal and sex chromosome abnormalities along with specific gene microdeletions and mutations have been described in patients with severe male factor infertility[59,60,61,62,63]. The most common genetic abnormality identified on genetic screening of infertile men is Klinefelter's syndrome 47,XXY with an occurrence of 1 in 500 live male births[1]. Klinefelter's syndrome is reported in 13% of cases of azoospermia[63]. Sperm are found in testicular biopsies from these patients in 40–50% of reported series[64,65] and fertilization rates greater than 50% using ICSI have been reported[66]. Preimplantation genetic diagnosis (PGD) is recommended by several investigators because it allows the couple the option to terminate a genetically abnormal embryo prior to transfer[64,66]. Despite an increased potential for genetically abnormal sperm, there have been several reports of both mosaic and nonmosaic Klinefelter patients having successful pregnancies and births of genetically normal children without PGD[67,68].

Microdeletions of the long arm of the Y chromosome have been extensively studied and identified at the molecular level. Both complete and partial deletions of the azoospermia factor (AZF) locus have been described and subclassified into several regions (AZFa, AZFb, AZFc); these deletions are associated with sertoli-cell only syndrome, maturation arrest, and hypospermatogenesis. The most common type of deletion is found on the AZFc region (60%); however, the worst prognosis is associated with AZFb deletions, resulting in the highest rate of completely absent spermatogenesis (100%)[69]. The incidence of Y chromosome microdeletions among infertile men varies between 1.5–10.8% in several reported international studies[61,62,63,70]. These deletions are extremely rare (0.7%) in patients with sperm concentrations greater than 5 million/ml[70]. Transmission of Y microdeletions could have implications in the offspring beyond male infertility. It has been reported that up to one-third of patients with Turner's stigmata and sexual ambiguity have associated Y microdeletions[71].

Cystic fibrosis (CF) represents the most common autosomal recessive disease in Caucasians with a 1 in 2,500 rate of disease and a 1 in 25 carrier rate reported in this population[72]. An association between CF and infertility is well established with 98% of CF men having congenital bilateral absence of the vas deferens (CBAVD) and resulting azoospermia[73]. Clinically, this Wolffian Duct defect results in absence of the vas deferens, seminal vesicles, and portions of the epididymis. Spermatogenesis is characteristically normal in these patients, and epididymal or testicular sperm is usually available for ICSI. Carriers of the CF mutation have varying degrees of reproductive tract anomalies as well. The diagnosis of CF has been facilitated ever since the discovery of the CF transmembrane conductance regulator (CFTR) gene and over 800 mutations have been described[74]. This gene identification has allowed the diagnosis of CF mutations in 72% of patients with CBAVD, 30% with CUAVD, and 34% with epididymal obstruction[72]. The obvious implications for reproduction in this population require that the female partners are also screened prior to any attempts of ICSI and appropriate genetic counseling given.

Genomic imprinting is the process of DNA methylation in germ cells that regulates the expression of a single parental gene line. This process occurs during gamete production and early embryo development[75]. ICSI is performed during critical times of imprinting, potentially interfering with gene expression. Beckwith-Wiedemann syndrome (BWS) and Angelman syndrome (AS) are both associated with imprinted gene clusters. Recent reports have implicated a higher rate of BWS and AS in children born after ICSI[76,77]. Further research into the relationship between genomic imprinting and ICSI is still needed.

## COSTS

The economic factors associated with ART are important considerations for any couple struggling with infertility. Most insurance carriers in the U.S. do not offer coverage for ART and there are currently few state-mandated regulations for this therapy[78]. The costs of ART must take numerous factors into consideration including the evaluation of the patient, the need for expensive fertility drugs to cause the eggs to mature, surgical intervention to retrieve the eggs, fertilizing and incubating the embryos, multiple gestation pregnancies, complications associated with preterm labor, prolonged hospitalization, retrieving and processing the sperm, and finally, the desire for future children. The success of each cycle of ART is equally important when considering the overall cost of this therapy. Taking the cost of most of these factors into account, Neumann et al. reported the average cost for a successful first cycle of conventional IVF to be $66,667[79]. In the best-case scenario (i.e., a female patient with tubal factor infertility), the cost was as low as $50,000 per delivery for the first cycle. However in the older female (over 40 years) coupled with a male-factor, the cost rose to $160,000 for the first cycle.

With the advent of ICSI, some people have even advocated bypassing reconstruction of the male reproductive tract and proceeding directly to MESA/TESE and IVF/ICSI in couples with obstructive azoospermia. Using epididymal or testicular sperm to perform ICSI has overcome the disappointing results seen with conventional IVF in the past[32]. The success of ICSI in this setting and the lack of a longer waiting period to maximize patency after vasectomy reversal has made IVF/ICSI a seemingly attractive alternative. However, when comparing the costs of vasectomy reversal to ICSI, the appeal quickly fades. In a recent review, the average cost per delivery with an initial surgical approach for vasectomy reversal was only $25,475 with a delivery rate of 47%. This is in contrast to the cost of sperm retrieval and ICSI at an average of $72,521 with a delivery rate of 33%[80]. These results were supported by another study from Germany demonstrating a cost per live birth for vasovasostomy (VV) of €2,793 compared to €14,547 for MESA/TESE and ICSI[81].

In some patients with a prolonged obstructive interval postvasectomy, VV is not an appropriate procedure to restore patency because of a secondary epididymal obstruction that may develop over time.

In this instance, an epididymovasostomy (EV) would be the recommended procedure. Kolettis and Thomas compared the costs of performing the technically more difficult EV to MESA with IVF/ICSI[4]. The average cost for EV was $31,099 with a delivery rate of 36% compared to $51,024 for an assumed delivery rate of 29% for MESA with IVF/ICSI. Other authors have looked at the cost of performing a repeat microscopic reconstruction after a previous failed attempt in comparison to proceeding to MESA and ICSI[82]. Even in this setting, the cost of MESA and ICSI was 2.4 times the charges per delivery obtained when performing repeat microscopic reconstruction, with a lower success rate per cycle[82].

It has been argued that for couples desiring vasectomy reversal with an older female partner, proceeding to MESA and ICSI is the treatment of choice. In a study of patients undergoing vasectomy reversal where the female partners were a median of 40 years old (range 38–48), Deck and Berger found that the comparative costs for microsurgical reconstruction and MESA/ICSI were significantly different ($28,530 vs. $103,940, respectively)[83]. The live birth rate was 17% in the vasectomy reversal group compared to 8% in a comparable cohort of women over 36 years old undergoing ICSI.

## CONCLUSION

The field of assisted reproductive technology (ART) has expanded significantly in the last 2 decades. The implementation of micromanipulation of oocytes has now allowed couples with even the most severe cases of male factor infertility to achieve their own genetic pregnancy. With a growing experience in performing IVF and ICSI, a better understanding of the factors necessary for ART success will develop and we will improve on the outcomes of this procedure. The U.S., along with several other countries, now has government regulations controlling the practice of ART as well as agencies that monitor the success of these procedures. Theses data are readily available to the savvy consumer of ART.

It is important to remember that the oldest ICSI baby is still only 13 years old. Our understanding about the risks associated with this procedure is becoming more apparent as we follow the longitudinal developmental milestones of these children. We are already aware of certain increased congenital abnormalities in these IVF/ICSI offspring. However, we are not yet certain of how these children will fare as they mature and enter adulthood, facing the superimposed impact of heart disease, cancer, and aging. The ultimate question of whether ICSI will impact their disease incidence or life expectancy is still to be determined.

Any couple considering IVF and ICSI should be made aware of the immediate and long-term costs associated with ICSI. Apart from the personal costs incurred by the couple during the procedure, there are societal costs to be considered for the growing numbers of children with their special needs who are siblings of multiple gestations. Additionally, when simply considering the costs of IVF/ICSI in lieu of surgical reconstruction of the male reproductive tract, there are very few instances where it is economically prudent to proceed with ART over surgical correction of the male partner.
